# Gain of 1q As a Prognostic Biomarker in Wilms Tumors (WTs) Treated With Preoperative Chemotherapy in the International Society of Paediatric Oncology (SIOP) WT 2001 Trial: A SIOP Renal Tumours Biology Consortium Study

**DOI:** 10.1200/JCO.2015.66.0001

**Published:** 2016-07-18

**Authors:** Tasnim Chagtai, Christina Zill, Linda Dainese, Jenny Wegert, Suvi Savola, Sergey Popov, William Mifsud, Gordan Vujanić, Neil Sebire, Yves Le Bouc, Peter F. Ambros, Leo Kager, Maureen J. O'Sullivan, Annick Blaise, Christophe Bergeron, Linda Holmquist Mengelbier, David Gisselsson, Marcel Kool, Godelieve A.M. Tytgat, Marry M. van den Heuvel-Eibrink, Norbert Graf, Harm van Tinteren, Aurore Coulomb, Manfred Gessler, Richard Dafydd Williams, Kathy Pritchard-Jones

**Affiliations:** Tasnim Chagtai, William Mifsud, Neil Sebire, Richard Dafydd Williams, and Kathy Pritchard-Jones, University College London Institute of Child Health, London; Sergey Popov, University Hospital of Wales; Gordan Vujanić, Cardiff University School of Medicine, Cardiff, United Kingdom; Christina Zill, Jenny Wegert, and Manfred Gessler, Wuerzburg University, Wuerzburg; Marcel Kool, German Cancer Research Center, Heidelberg; Norbert Graf, Saarland University Hospital, Homburg, Germany; Linda Dainese, Yves Le Bouc, Annick Blaise, and Aurore Coulomb, Sorbonne Universités; Linda Dainese, Yves Le Bouc, and Aurore Coulomb, Assistance Publique Hôpitaux de Paris–Hôpital Armand Trousseau, Paris; Christophe Bergeron, Centre Léon Bérard, Lyon, France; Suvi Savola, MRC-Holland; Harm van Tinteren, Netherlands Cancer Institute, Amsterdam; Godelieve A.M. Tytgat and Marry M. van den Heuvel-Eibrink, Princess Maxima Center for Pediatric Oncology/Hematology, Utrecht, the Netherlands; Peter F. Ambros and Leo Kager, Children's Cancer Research Institute; Leo Kager, St Anna Children's Hospital, Vienna, Austria; Maureen J. O'Sullivan, Our Lady's Children's Hospital, Dublin, Ireland; and Linda Holmquist Mengelbier and David Gisselsson, Lund University, Lund, Sweden.

## Abstract

**Purpose:**

Wilms tumor (WT) is the most common pediatric renal tumor. Treatment planning under International Society of Paediatric Oncology (SIOP) protocols is based on staging and histologic assessment of response to preoperative chemotherapy. Despite high overall survival (OS), many relapses occur in patients without specific risk factors, and many successfully treated patients are exposed to treatments with significant risks of late effects. To investigate whether molecular biomarkers could improve risk stratification, we assessed 1q status and other potential copy number biomarkers in a large WT series.

**Materials and Methods:**

WT nephrectomy samples from 586 SIOP WT 2001 patients were analyzed using a multiplex ligation-dependent probe amplification (MLPA) assay that measured the copy number of 1q and other regions of interest.

**Results:**

One hundred sixty-seven (28%) of 586 WTs had 1q gain. Five-year event-free survival (EFS) was 75.0% in patients with 1q gain (95% CI, 68.5% to 82.0%) and 88.2% in patients without gain (95% CI, 85.0% to 91.4%). OS was 88.4% with gain (95% CI, 83.5% to 93.6%) and 94.4% without gain (95% CI, 92.1% to 96.7%). In univariable analysis, 1q gain was associated with poorer EFS (*P* < .001; hazard ratio, 2.33) and OS (*P* = .01; hazard ratio, 2.16). The association of 1q gain with poorer EFS retained significance in multivariable analysis adjusted for 1p and 16q loss, sex, stage, age, and histologic risk group. Gain of 1q remained associated with poorer EFS in tumor subsets limited to either intermediate-risk localized disease or nonanaplastic localized disease. Other notable aberrations associated with poorer EFS included *MYCN* gain and *TP53* loss.

**Conclusion:**

Gain of 1q is a potentially valuable prognostic biomarker in WT, in addition to histologic response to preoperative chemotherapy and tumor stage.

## INTRODUCTION

Wilms tumor (WT) is the most common childhood renal malignancy.^1^ Most patients are treated effectively, with approximately 90% achieving 5-year survival, but new approaches are needed to improve the outcome of the remainder, especially in cases of recurrence, where only approximately 50% will survive.^2,3^ More specific biomarkers for treatment stratification could also reduce the therapeutic burden on the successfully treated majority. Treatment planning is currently determined by clinical staging and histopathologic criteria. In countries that follow the protocols of the International Society of Paediatric Oncology (SIOP), patients with WT typically receive neoadjuvant chemotherapy, and the histopathology at nephrectomy is used to classify patients into risk groups. Tumors with diffuse anaplasia or that contain a high proportion of chemoresistant blastema (blastemal type) are regarded as high risk; epithelial, stromal, mixed, and regressive subtypes are classed as intermediate risk, and completely necrotic tumors are classed as low risk.^4^ Using this classification, the SIOP WT 2001 trial recently reported that doxorubicin can be safely omitted from the treatment of stage II to III intermediate-risk histology tumors, although it still adds benefit when patients have high-risk histology.^5,6^ However, high-risk tumors are relatively uncommon, and most relapses still occur in patients with localized (stage I to III) low- and intermediate-risk histology tumors. Therefore, there is a clinical need to improve the sensitivity and specificity of risk prediction in WT. The SIOP WT 2001 trial included, as a secondary aim, investigation of the potential value of including molecular biomarkers in addition to the current use of tumor stage and histology in risk stratification.

Previous analyses have identified multiple recurrent aberrations in WT. Notable genes with documented mutations include *WT1*,^7-9^ *CTNNB1*,^10^ *WTX* (*AMER1*),^11^ *TP53*,^12^ *FBXW7*,^13^ *MYCN*, *SIX1/2*, *DICER1*, *DROSHA*, and *DGCR8*.^14-18^ Copy neutral loss of heterozygosity on 11p, common in stromal-type tumors, can lead to both second hit inactivation of mutated *WT1* on 11p13 and aberrant expression of the imprinted genes *H19* and *IGF2* on 11p15; the latter locus is also frequently targeted by epigenetic abnormalities.^19^ Several WT genes, including *WT1*, *WTX*, *TP53*, *FBXW7*, and *MYCN* are also subject to recurrent copy number aberrations, as are a number of larger-scale genomic regions, but few of these are of known prognostic relevance. Simultaneous allele loss of 1p and 16q is associated with adverse outcome in patients with favorable-histology WT treated with immediate nephrectomy, and this biomarker is already used in treatment stratification by the Children's Oncology Group of North America.^20^ We have recently shown that *TP53* mutation and 17p loss, aberrations largely confined to anaplastic histology WT, are potential adverse indicators within this subtype.^21^ However, the utility of both these biomarkers is limited by their relative rarity. Genomic gain of 1q, one of the most common copy number changes in WT,^22-25^ seems to be associated with poor outcome, as is gain of *MYCN*.^18^ Recent studies in the United States and United Kingdom have focused on the significance of 1q gain and support its prognostic value.^26,27^

The principal aim of this study was to assess the feasibility of using 1q gain as a prognostic biomarker by determining its association with event-free survival (EFS) and overall survival (OS) in a cohort drawn entirely from the SIOP WT 2001 clinical trial (which is, to our knowledge, the largest SIOP cohort so far analyzed for this biomarker). Accordingly, a rapid and relatively low-cost multiplex ligation-dependent probe amplification (MLPA) assay^28^ was developed and optimized to assess the copy number status of 1q and other key regions or gene-specific loci, including 1p, 16q, *WT1*, *WTX*, *TP53*, *MYCN,* and *FBXW7*.

## MATERIALS AND METHODS

### Patients

Patients registered prospectively in the SIOP WT 2001 clinical trial and treated with preoperative chemotherapy according to standardized risk-stratified regimens on the basis of tumor stage, histology, and metastatic response to preoperative chemotherapy^5,29^ with stage I to IV WT and available frozen tumor were eligible for this study. Selection criteria and patient characteristics are provided in the Data Supplement (Methods). Informed consent was obtained from all families. Our research was approved by local ethics committees and conducted in accordance with the Helsinki Declaration.

### Samples

All samples were freshly frozen specimens obtained at nephrectomy. Genomic DNA was prepared by standard methods. Only WT with a tumor content ≥ 50% as determined by a pediatric pathologist were used for this study (N = 586; Data Supplement Table S1). Full details, including sample inclusion criteria and DNA quality control (QC) metrics, are listed in the Data Supplement.

### MLPA

The MLPA assay (P380-X2) was designed and developed in collaboration with MRC-Holland (Amsterdam, the Netherlands). The panel included 33 probes for regions or genes of interest, including seven on 1p, five on 1q, six on 16q, and three each targeting *MYCN* (2p), *TP53* (17p), *FBXW7* (4q), *WT1* (11p), and *WTX* (*AMER1*, Xq), as well as reference and QC probes (Data Supplement Table S2). MLPA reactions were performed according to the manufacturer’s instructions, with appropriate internal quality and external normal controls. Polymerase chain reaction products were analyzed on an ABI 3730 DNA Analyzer, (Thermo Fisher Scientific, Waltham, MA).

### Data Analysis

**Fig 1.**
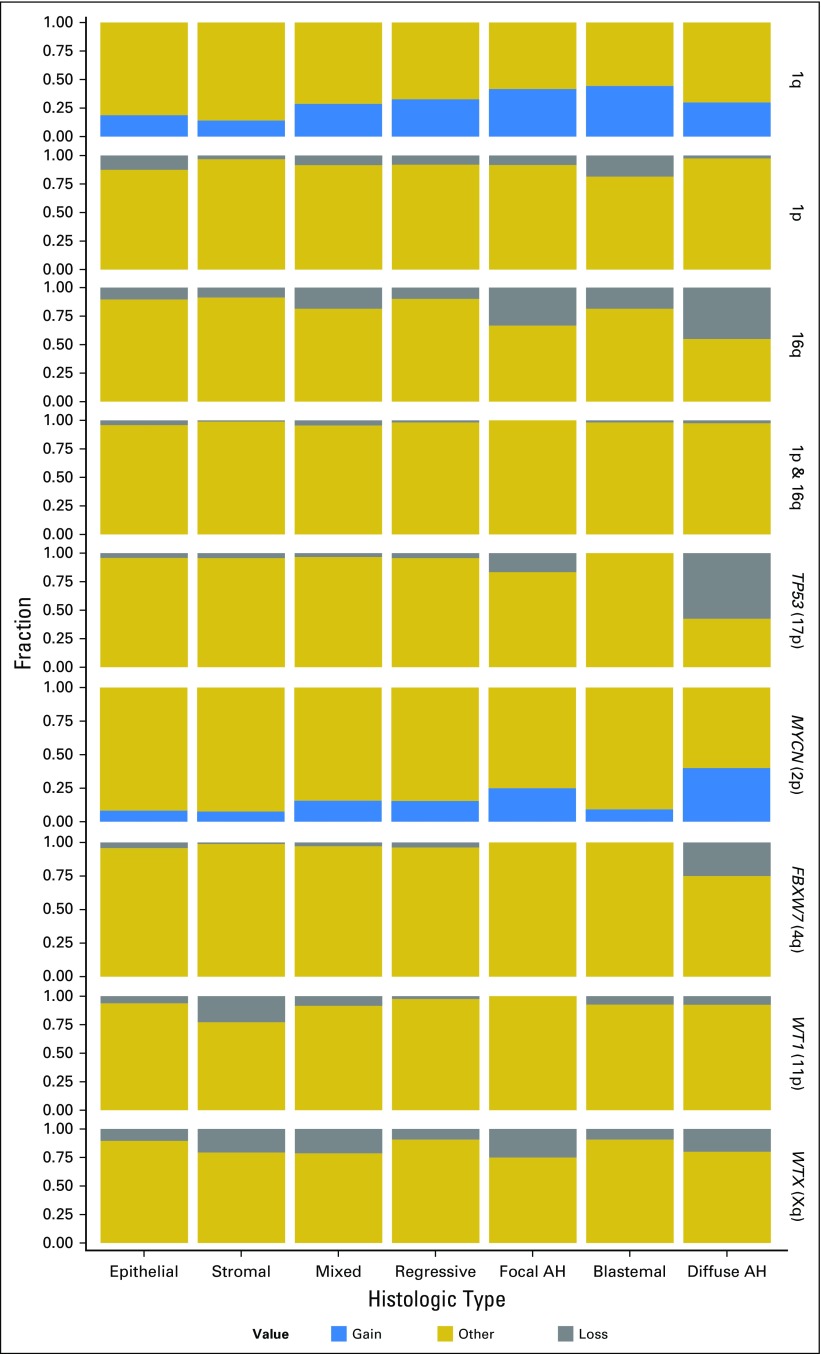
Aberration frequency histograms for loci of interest in specific histologic subtypes of Wilms tumor (full series, N = 586). AH, anaplastic histology.

**Fig 2.**
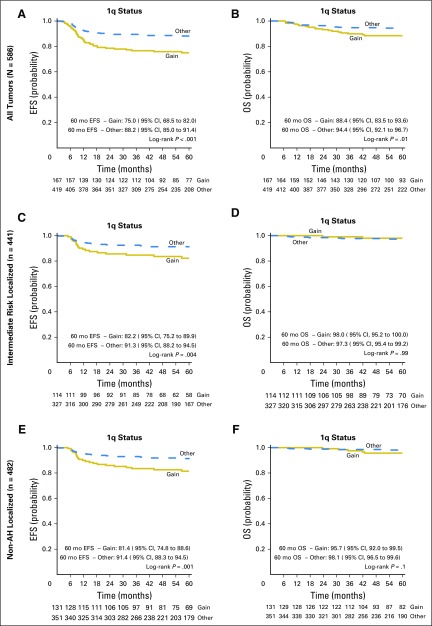
(A, C, E) Event-free (EFS) and (B, D, F) overall survival (OS) curves for (A, B) complete series, (C, D) intermediate-risk localized disease, and (E, F) nonanaplastic localized disease Wilms tumors, stratified by 1q status. AH, anaplastic histology.

Copy number ratios relative to the normal reference were calculated with Coffalyser.NET software (MRC-Holland) using the default settings. A numerical gain was scored when the ratios exceeded 1.2 and a loss when the ratios were lower than 0.8; all other values were considered to be normal diploid. For individual genes, aberrations were scored by the median ratio of the gene-specific probes. For 1p, 1q, and 16q, a gain or loss of at least two consecutive probed loci was required to score a chromosome arm aberration. Associations between copy number aberrations and histopathologic subtypes (Fig 1) were calculated by logistic regression, and survival analyses (Table 1; Fig 2; Data Supplement) were performed using the Kaplan-Meier estimator, log-rank test, and Cox proportional hazards regression model (Data Supplement Methods). For multivariable analyses, the factors considered are listed in the “Variable” column of Table 2.

**Table 1.** Univariable Survival Analyses

Patient Series	Aberration	No. of Patients	No. of Relapses	Event *P*	Event HR	5-Year EFS	No. of Deaths	Death *P*	Death HR	5-Year OS
Unselected patients	1q gain	167	43	< .001	2.33	75	19	.01	2.16	88.4
(N = 586)	1q other	419	49			88.2	22			94.4
	1p loss	49	11	.17	1.55	77.9	5	.38	1.52	89
	1p other	537	81			85	36			93
	16q loss	94	20	.12	1.48	78.5	11	.07	1.88	89.1
	16q other	492	72			85.5	30			93.3
	1p and 16q loss	16	3	.76	1.22	81.2	0	.27	0.01	100
	1p and 16q other	570	89			84.5	41			92.4
	*TP53* (17p) loss	44	19	< .001	4.03	55.2	16	< .001	9.80	63.7
	*TP53* (17p) other	542	73			86.7	25			94.9
	*WT1* (11p) loss	50	6	.45	0.73	86.6	1	.15	0.26	97.8
	*WT1* (11p) other	536	86			84.2	40			92.2
	*WTX* (Xq) loss	93	10	.13	0.61	91.4	2	.04	0.26	97.8
	*WTX* (Xq) other	493	82			83	39			91.6
	*MYCN* (2p) gain	88	26	< .001	2.45	71.2	14	< .001	3.09	83.7
	*MYCN* (2p) other	498	66			86.7	27			94.2
	*MYCN* (only) gain	60	20	< .001	2.72	67.9	12	< .001	3.91	79.4
	*MYCN* (only) other	526	72			86.3	29			94.2
	*FBXW7* (4q) loss	24	15	< .001	6.58	38	10	< .001	9.62	59.3
	*FBXW7* (4q) other	562	77			86.4	31			94
IR stage I-III	1q gain	114	22	.004	2.21	82.2	3	.99	1.01	98
(n = 441)	1q other	327	29			91.3	8			97.3
	1p loss	34	6	.27	1.61	83.7	1	.88	1.17	97
	1p other	407	45			89.4	10			97.5
	16q loss	59	9	.4	1.36	84.6	3	.21	2.27	96.4
	16q other	382	42			89.5	8			97.6
	1p and 16q loss	13	2	.69	1.33	84.6	0	.56	0.01	100
	1p and 16q other	428	49			89	11			97.4
	*TP53* (17p) loss	19	6	.004	3.23	67.4	3	< .001	8.33	88.2
	*TP53* (17p) other	422	45			89.9	8			97.8
	*WT1* (11p) loss	42	5	.94	1.03	86.3	0	.29	0.00	100
	*WT1* (11p) other	399	46			89.2	11			97.2
	*WTX* (Xq) loss	79	8	.58	0.81	92.4	0	.11	0.00	100
	*WTX* (Xq) other	362	43			88.1	11			96.9
	*MYCN* (2p) gain	61	14	.003	2.49	78.2	4	.03	3.50	93.4
	*MYCN* (2p) other	380	37			90.7	7			98.1
	*MYCN* (only) gain	42	11	.001	2.86	75.7	4	.002	5.49	90.3
	*MYCN* (only) other	399	40			90.3	7			98.2
	*FBXW7* (4q) loss	13	6	< .001	4.85	59.3	1	.23	3.25	100
	*FBXW7* (4q) other	428	45			89.9	10			97.4
Non-AH stage I-III	1q gain	131	26	.001	2.34	81.4	6	.1	2.48	95.7
(n = 482)	1q other	351	30			91.4	6			98.1
	1p loss	42	8	.13	1.78	82	3	.05	3.47	92.3
	1p other	440	48			89.3	9			97.9
	16q loss	64	9	.59	1.22	85.7	2	.8	1.22	98.4
	16q other	418	47			89	10			97.2
	1p and 16q loss	14	2	.76	1.25	85.7	0	.55	0.01	100
	1p and 16q other	468	54			88.7	12			97.3
	*TP53* (17p) loss	17	5	.02	2.82	69.3	2	.02	5.24	93.3
	*TP53* (17p) other	465	51			89.3	10			97.5
	*WT1* (11p) loss	45	5	.9	0.94	87.4	0	.27	0.00	100
	*WT1* (11p) other	437	51			88.7	12			97.1
	*WTX* (Xq) loss	81	8	.5	0.77	92.6	0	.1	0.00	100
	*WTX* (Xq) other	401	48			87.7	12			96.8
	*MYCN* (2p) gain	63	14	.01	2.30	78.9	4	.04	3.23	93.4
	*MYCN* (2p) other	419	42			90.1	8			98
	*MYCN* (only) gain	43	11	.002	2.68	76.2	4	.003	5.10	90.3
	*MYCN* (only) other	439	45			89.8	8			98.1
	*FBXW7* (4q) loss	13	6	< .001	4.83	59.3	1	.23	3.28	100
	*FBXW7* (4q) other	469	50			89.5	11			97.3

Abbreviations: AH, anaplastic histology; EFS, event-free survival; HR, hazard ratio; IR, intermediate risk; OS, overall survival.

**Table 2.** Multivariable Survival Analyses

Patient Series	Variable	Comparison	Event-Free Survival				Overall Survival			
			*P*	HR	Lower	Upper	*P*	HR	Lower	Upper
Unselected patients (n = 585)	1p loss	No loss	.95	0.98	0.5	1.91	.45	0.67	0.24	1.89
	1q gain	No gain	.002	1.98	1.27	3.07	.16	1.61	0.83	3.15
	16q loss	No loss	.63	1.14	0.68	1.91	.39	1.37	0.67	2.83
	Female	Male	.98	0.99	0.65	1.51	.81	0.93	0.49	1.74
	Stage II	Stage I	.43	1.27	0.71	2.27	.06	3.13	0.96	10.26
	Stage III	Stage I	.17	1.52	0.83	2.79	.01	4.39	1.36	14.12
	Stage IV	Stage I	< .001	4.58	2.58	8.15	< .001	21.65	6.93	67.66
	High risk	Intermediate risk	.001	2.28	1.41	3.68	< .001	8.13	4.05	16.32
	Age	Per unit	.06	1.01	1	1.01	.48	1	0.99	1.01
IR stage I-III (n = 440)	1p loss	No loss	.97	1.02	0.41	2.5	.84	0.81	0.1	6.74
	1q gain	No gain	.04	1.92	1.05	3.51	.44	0.56	0.13	2.42
	16q loss	No loss	.64	1.2	0.56	2.55	.09	3.51	0.82	15.12
	Female	Male	.27	0.73	0.41	1.28	.04	0.24	0.06	0.92
	Stage II	Stage I	.76	1.11	0.57	2.18	.13	3.25	0.71	14.82
	Stage III	Stage I	.13	1.73	0.85	3.54	.02	7.01	1.45	33.78
	Age	Per unit	.32	1	1	1.01	.71	1	0.99	1.02
Non-AH stage I-III (n = 481)	1p loss	No loss	.73	1.15	0.52	2.54	.45	1.75	0.4	7.61
	1q gain	No gain	.02	2	1.13	3.57	.62	1.39	0.38	5.11
	16q loss	No loss	.96	0.98	0.46	2.06	.77	1.27	0.26	6.29
	Female	Male	.33	0.77	0.45	1.31	.04	0.24	0.06	0.93
	Stage II	Stage I	.36	1.34	0.72	2.5	.05	5.12	0.99	26.4
	Stage III	Stage I	.26	1.51	0.74	3.08	.06	5.58	0.91	34.08
	High risk	Intermediate risk	.71	0.85	0.38	1.94	.45	1.71	0.42	7
	Age	Per unit	.11	1.01	1	1.01	.53	1.01	0.99	1.02

Abbreviations: AH, anaplastic histology; HR, hazard ratio; IR, intermediate risk.

## RESULTS

### Sample Series and Histologic Subtypes

A total of 586 patients with stages I to IV WTs, in which tumor content was confirmed by histologic review, high-quality DNA was successfully extracted, and data exceeded QC thresholds (Data Supplement Methods), were included in the analysis. In this series (Data Supplement Table S1), median clinical follow-up was 68 months, 92 patients had an event (relapse), and 41 patients died. In 55% of tumors (321 of 586), at least one of the major copy number aberrations targeted by the assay (1q gain, 1p loss, 16q loss, *MYCN* gain, *TP53* loss, *WT1* loss, *WTX* loss, or *FBXW7* loss) was detected (Data Supplement Table S1). Overall, the numbers of alterations identified across all markers were consistent with previous reports. Some aberrations were more common in specific subtypes (Fig 1; Data Supplement Table S3) and some significant associations were noted. Compared with mixed-type histology, diffuse anaplasia was significantly associated with *TP53* (17p) loss (*P* < .001), *MYCN* (2p) gain (*P* < .001), 16q loss (*P* < .001), and *FBXW7* (4q) loss (*P* < .001), the latter presumably reflecting an association between anaplasia and whole-arm 4q loss, which we have described previously.^30^ The stromal subtype was associated with *WT1* (11p) loss (*P* = .0014), consistent with previous reports, and with a significantly lower frequency of 1q gain than the other subtypes (*P* = .00912). A gain of 1q was most frequent in blastemal-type tumors (Fig 1), but not to a statistically significant extent. We also noted an association between the regressive type and a lower frequency of *WTX* (*AMER1*, Xq) loss. Most aberrations, including 1q gain, were somewhat less common in stage I than in higher stage tumors (Data Supplement Table S4).

### Univariable Outcome Analysis of 1q Gain

In the complete series of 586 patients (Table 1; Figs 2A and 2B), 167 tumors (28.5%) had 1q gain. Five-year EFS in the 1q-gain group was 75.0% (95% CI, 68.5% to 82.0%) and 88.2% in the no-gain group (95% CI, 85.0% to 91.4%). The corresponding OS values were 88.4% (95% CI, 83.5% to 93.6%) and 94.4% (95% CI, 92.1% to 96.7%), respectively. At the alpha significance level of .05, univariable analyses using the Cox proportional hazards regression model showed that 1q gain was associated with poorer EFS (hazard ratio [HR], 2.33; log-rank *P* < .001) and OS (HR, 2.16; *P* = .01).

Because 1q gain as a potential biomarker would be of most value in optimizing risk stratification in localized tumors, we also considered two important subsets. The first consisted of 441 patients with localized disease (stage I to III), intermediate-risk histology tumors according to the SIOP classification. In univariable analysis (Table 1; Figs 2C and 2D), 1q gain was significantly associated with inferior EFS (*P* = .004; HR, 2.21) but not OS (*P* = .99; HR, 1.01). The second subset was selected to allow direct comparison with the Children’s Oncology Group risk stratification. Among 482 patients with localized, nonanaplastic tumors (ie, excluding both diffuse and focal anaplastic but including blastemal-type WTs), 1q gain was associated with poorer EFS (*P* = .001; HR, 2.34) but not OS (*P* = .1; HR, 2.48; Table 1; Figs 2E and 2F).

### Univariable Outcome Analysis of 1p Loss and 16q Loss

Neither 1p loss nor 16q loss, nor combined loss of 1p and 16q, considered as a single biomarker in a univariable Cox model, was significantly associated with EFS or OS in the entire tumor series at the *P* = .05 level (Data Supplement). This was also true for the subsets, with the single exception of a marginal association between 1p loss and poorer OS in nonanaplastic patients (Table 1; Data Supplement Figs S1, S2, and S3).

### Multivariable Outcome Analyses

In a multivariable outcome analysis including 1q gain, 1p loss, 16q loss, tumor stage and histologic risk group, sex, and age, 1q gain was significantly associated with poorer EFS (HR, 1.98; *P* = .002), but not OS (HR, 1.61; *P* = .16; Table 2). The only other independent factors of those assessed for adverse outcome in the full series (N = 586) were high-risk histology and stage IV disease. The significant independent association of 1q gain with adverse EFS but not OS persisted in the subsets of intermediate-risk histology, localized WT (n = 440; EFS HR, 1.92; *P* = .04) and nonanaplastic, localized WT (n = 481; EFS HR, 2.0; *P* = .02).

### Univariable Analysis of Gene-Specific Markers

The outcome data for the other markers covered by the assay were also analyzed on an exploratory basis (Table 1; Data Supplement Figs S4-S9). *MYCN* (2p) gain was significantly associated with poorer EFS and OS in the complete data set, in the localized disease intermediate-risk subset, and in the localized disease subset with anaplastic WTs excluded (Data Supplement Fig S4). Using a more specific definition of *MYCN* gain, *MYCN*-only gain (excluding from the *MYCN*-gain group those tumors in which the *DYSF* control probe on 2p was also gained, because gains at both loci were likely to be whole-arm gains), we saw higher HRs and lower *P* values (Table 1; Data Supplement Fig S5). Similarly, *TP53* (17p) loss was significantly associated with inferior EFS and OS in the complete series and, perhaps surprisingly, in both subsets, neither of which included diffuse anaplastic WTs (Table 1; Data Supplement Fig S6).

A third copy number change, loss of the *FBXW7* locus on 4q, was significantly associated with poorer EFS and OS in the complete 586 tumor series, but only with poorer EFS in both subsets (Table 1; Data Supplement Fig S7). No significant associations were noted between the copy number status of *WT1* and outcome at the *P* = .05 significance level (Table 1; Data Supplement Fig S8). For *WTX*, there was no significant association with EFS, but improved OS was marginally associated with copy number loss in the complete series only (*P* = .04; Data Supplement Fig S9).

## DISCUSSION

This is, to our knowledge, the first study to carry out a large-scale analysis of 1q copy number aberrations in WT sampled at nephrectomy after neoadjuvant chemotherapy according to the SIOP WT 2001 protocol. The clinical characteristics of the patient cohort were representative of the entire registered population who had received preoperative chemotherapy and presented with unilateral disease; 586 patients with stage I to IV WT, including all intermediate- and high-risk histologic subtypes, were analyzed. We found that 1q gain is significantly associated with poorer EFS and OS in univariable analyses, with HRs in excess of two-fold for relapse and death. These results are broadly consistent with those recently reported in a study of patients treated by immediate nephrectomy under Children's Oncology Group protocols without preoperative chemotherapy^26^ and, although it is essential to assess 1q gain independently in cohorts treated under both regimens, it is encouraging to note that it seems to be a prognostically valuable marker regardless of treatment protocol. However, in our multivariable analysis of the SIOP data, which also considered 1p loss, 16q loss, sex, stage, age, and histologic risk group, 1q gain remained significantly associated only with EFS (HR, 1.98; *P* = .002) and not OS (HR, 1.61; *P* = .16). This lack of association with OS is perhaps not surprising, given the comparatively low number of deaths in the patient series (41, compared with 92 relapses), reflecting the relative success of second-line therapy.

Because just over half of all relapses occur in children with localized WT that are not of high-risk histology, we analyzed this subset of patients (n = 441) in which treatment intensification to reduce relapse risk would be clinically appropriate and feasible. Here, we found that 1q gain retained its independent prognostic significance for EFS (HR, 1.92; *P* = .04) but not OS in multivariable analysis. Similar results (HR, 2.00; *P* = .02) were obtained for localized nonanaplastic tumors (n = 481), excluding both diffuse and focal anaplastic WTs but retaining blastemal type. This subset is comparable to the current North American definition of favorable histology for localized patients treated by immediate nephrectomy (where blastemal type, which implies chemoresistance, cannot be defined).

In contrast to a previous report on immediate nephrectomy patients,^20^ we did not find that the combination of 1p loss and 16q loss was prognostically significant in the SIOP series in the univariable or multivariable analyses. This was true for both EFS and OS, in the entire series, and in the nonanaplastic and intermediate-risk subsets. However, the size of our sample series (significantly smaller than the immediate nephrectomy cohort) meant that the current study did not have sufficient power to assess reliably the prognostic significance of relatively rare aberrations such as combined 1p and 16q loss, observed in only 16 patients (three of whom relapsed). We note also that any copy neutral loss of heterozygosity, another possible mechanism of allele loss at these loci, would not be detected by MLPA. A substantially larger series would be required to obtain definitive results for this rare combined marker in SIOP patients.

In a previous study,^18^ we presented an analysis of *MYCN* copy number status that included 234 of the samples described in this study. Therefore, our observations are not independent, but the current expanded series should give a more reliable indication of the prognostic relevance of *MYCN* gain. As before, we note that *MYCN* gain seems to be a promising adverse prognostic indicator for WT, for both EFS and OS (Data Supplement Fig S4). We also analyzed the data using a more specific definition of gain (*MYCN*-only gain; Data Supplement Fig S5), excluding whole-arm gains. The adverse association with both EFS and OS was retained in all univariable analyses, but with lower *P* values and higher HRs throughout, perhaps suggesting that the type of genomic disruption that has given rise to *MYCN* gain, rather than the relative dose of *MYCN* with regard to the genomic baseline, is more prognostically relevant. A higher resolution (eg, single nucleotide polymorphism array) platform that allows precise delineation of the region of gain and distinguishes between focal events, such as those we described previously,^13^ and larger segmental changes would allow us to address this question.

In a previous study we described an association between poor outcome and *TP53* aberrations (typically point mutation coincident with whole-arm copy number loss of 17p) in diffuse anaplastic tumors.^21^ Interestingly, *TP53* (17p) loss in the current study was associated with poorer EFS and OS, even in the subsets that excluded anaplastic tumors. It is currently not known whether the nonanaplastic tumors with copy number loss at this locus also had *TP53* mutations or whether these tumors had any unusual histologic features, such as nuclear unrest.^31^

Loss of the *FBXW7* locus on 4q was significantly associated with poorer EFS and OS in the complete tumor series and with adverse EFS only in the subsets. In earlier studies, we reported focal homozygous loss and point mutation of *FBXW7* in several intermediate-risk histology WTs,^13^ as well as broader but typically single copy loss of 4q associated with anaplasia^30^; the current assay does not distinguish between these types of aberrations.

Optimizing treatment to minimize the risk of long-term adverse effects without compromising EFS or OS is a principal aim of clinical research in WT. The previous SIOP randomized trial^5,6^ showed that therapeutic intensity could be reduced in patients with localized intermediate-risk tumors without affecting OS, at the cost of a 4.4% reduction in EFS (95% CI, 0.4% to 9.3%). Because it is clearly desirable for patients to avoid even treatable relapses, further refinement of first-line therapy remains a priority, and novel biomarkers may provide the key data required to improve risk stratification and maximize EFS. In this study, we have shown that MLPA provides a rapid and effective means of determining the status of copy number aberrations associated with poorer EFS. The relatively high frequency of 1q gain makes this marker particularly attractive for potential use in risk stratification. However, any change in intensity on the basis of 1q status alone would affect a significant proportion of patients who have experienced a reasonably good EFS when treated with current therapies and where relapse is salvageable. Hence, the SIOP Renal Tumours Study Group considers it may be more appropriate to define risk groups for treatment stratification on the basis of several combined molecular biomarkers, taking account of our findings of the adverse significance of *MYCN* gain and *TP53* loss and incorporating mutations in recently discovered WT genes, some of which are reported to have prognostic significance. This requires a prospective clinical study powered to include tumor stage and histologic risk group, both individually significant in our multivariable analysis, alongside quantitative assessment of the volume of blastema that survives preoperative chemotherapy, a further potential prognostic factor.^32^ This prospective study will also incorporate multiple sampling of each WT to determine the extent of intratumoral heterogeneity of 1q gain and other biomarkers. It will register all patients with a newly diagnosed renal tumor and continue the risk stratification and treatment arms for localized WT used in the SIOP WT 2001 trial. The study will be known as UMBRELLA, and is expected to open in 2016.
